# *Entamoeba histolytica*-induced NETs are highly cytotoxic on hepatic and colonic cells due to serine proteases and myeloperoxidase activities

**DOI:** 10.3389/fimmu.2024.1493946

**Published:** 2024-12-02

**Authors:** Fabian Jorge-Rosas, César Díaz-Godínez, Samuel García-Aguirre, Santiago Martínez-Calvillo, Julio César Carrero

**Affiliations:** ^1^ Departamento de Inmunología, Instituto de Investigaciones Biomédicas, Universidad Nacional Autónoma de México (UNAM), Ciudad de México, Mexico; ^2^ Unidad de Biomedicina, Facultad de Estudios Superiores Iztacala, Universidad Nacional Autónoma de México, Tlalnepantla, EM, Mexico

**Keywords:** *Entamoeba histolytica*, NETs, neutrophils, cell damage, HCT 116, Hep G2

## Abstract

During intestinal and liver invasion by the protozoan parasite *Entamoeba histolytica*, extensive tissue destruction linked to large neutrophil infiltrates is observed. It has been proposed that microbicidal components of neutrophils are responsible for the damage, however, the mechanism by which they are released and act in the extracellular space remains unknown. In previous studies, we have shown that *E. histolytica* trophozoites induce NET formation, leading to the release of neutrophil granule content into extruded DNA. In this work, we evaluate the possible participation of NETs in the development of amoeba-associated pathology and analyze the contribution of anti-microbial components of the associated granules. *E. histolytica-*induced NETs were isolated and their effect on the viability and integrity of HCT 116 colonic and Hep G2 liver cultures were evaluated. The results showed that simple incubation of cell monolayers with purified NETs for 24 h resulted in cell detachment and death in a dose-dependent manner. The effect was thermolabile and correlated with the amount of DNA and protein present in NETs. Pretreatment of NETs with specific inhibitors of some microbicidal components suggested that serine proteases, are mostly responsible for the damage caused by NETs on HCT 116 cells, while the MPO activity was the most related to Hep G2 cells damage. Our study also points to a very important role of DNA as a scaffold for the activity of these proteins. We show evidence of the development of NETs in amoebic liver abscesses in hamsters as a preamble to evaluate their participation in tissue damage. In conclusion, these studies demonstrate that amoebic-induced NETs have potent cytotoxic effects on target cells and, therefore, may be responsible for the intense damage associated with tissue invasion by this parasite.

## 1 Introduction

In humans, *Entamoeba histolytica* is the protozoan parasite responsible for amebiasis, an intestinal and sometimes extra-intestinal disease whose main clinical manifestations are dysentery and liver abscesses, respectively. This disease is common in developing countries in tropical areas with high levels of poverty and limited sanitation, including Africa, India and Latin American countries, mainly Mexico (1, 2). In this country, the National Epidemiological Surveillance System registered almost 9 million cases of amebiasis in the first decade of the 20th century (3). Amebiasis, therefore, represents a serious health problem that warrants various intervention strategies for its control.

The parasite infects the host when cysts are ingested in water or food contaminated with feces, and amebic invasion occurs in the intestine when the parasite reaches the trophozoite stage (4). When the amoeba penetrates the tissues, an intense inflammatory response occurs, characterized by massive recruitment of neutrophils, which seems to mitigate histological lesions and parasitic load in partially resistant murine models of intestinal and hepatic amebiasis (5, 6). However, in the highly susceptible golden hamster model, controlling inflammation with radiation or immunosuppressants inhibited the development of amoebic liver abscesses, suggesting a role for inflammatory cells, and in particular neutrophils, in amebic pathology (7–9). Thus, the role of neutrophils during invasive amebiasis is controversial, with dual behavior in the pathophysiology of amebiasis depending on the study model.

For many years, it has been speculated that the amoebicidal activity of neutrophils relies on the degranulation of primary/secondary granules (10, 11) and the generation of reactive oxygen species (ROS) (12). More recently, the formation of neutrophil extracellular traps (NETs) has been added to this arsenal, as neutrophils respond quickly and explosively by firing NETs at pathogenic *E. histolytica* trophozoites but not at the non-pathogenic *Entamoeba dispar* (13, 14). NETs are DNA meshes decorated with histones, serine proteases, oxidizing enzymes, and antimicrobial peptides that act as antimicrobial webs (15). Although there is evidence that NETs trap amoebae *in vitro* (14, 16–18), there is still controversy over whether they directly kill them (14, 19) or affect their infective capacity (13).

The most compelling evidence of the role of NETs in host defense comes from patients with chronic granulomatous disease (CGD) who lack a functional NADPH oxidase, indispensable for NET formation (20). Patients with CGD are highly susceptible to aspergillosis, but when NADPH oxidase function is restored by gene therapy, they acquired a protective profile against *Aspergillus nidulans* (20). Further evidence comes from knockout mice deficient in PAD4 (another important element for NET formation) which are more vulnerable to shigellosis than mice carrying the gene (21). Subsequent studies found that NET formation is a selective process apparently mediated by the pathogens size (22). Under these premises, protozoan parasites were soon identified as potent inducers of NETosis (23, 24). To date, *in vivo* and *in vitro* microbicidal activity of human NETs against protozoan parasites has been reported in *Leishmania amazonensis* (25), *Leishmania braziliensis* (26), *Trichomonas vaginalis* (27), and *Toxoplasma gondii* (28) models, suggesting that NETs are protective against these parasitic infections. However, the involvement of NETs in defense against *E. histolytica* remains controversial (13, 14, 19).

On the other hand, when the balance between the formation and degradation of NETs is altered by their exacerbated production or by deficiencies in DNA clearance systems, the spatiotemporal persistence of the networks in tissues leads to the development of disorders related to the pathological properties of the constituents of the NETs (29–31). This phenomenon is exemplified in patients with systemic lupus erythematosus (SLE), where autoreactive antibodies targeting NET nucleic acids form complexes that promote the production of additional NETs, resulting in a vicious cycle that exacerbates inflammation (32). During rheumatoid arthritis, NET histones undergo post-translational modifications (proteolytic cleavages, acetylation, citrullination, carbamylation, etc.), turning them a potential source of extracellular autoantigens (29, 33). Although the detrimental effect of NETs was initially observed only in autoimmune pathologies, new evidence suggests that they could also damage tissues and organs during infectious diseases such as sepsis (34), candidiasis (35), or COVID-19 (36). NETs have also been linked to the pathology of some parasitic infections, such as malaria by *Plasmodium falciparum* in children (37), their amount in circulation has been associated to the severity of the malaria in adults (38), and their release during the bovine infection with *Besnoitia besnoiti* has been demonstrated that cause damage to endothelial cells (39). More information on the role of NETs in parasitic infections by protozoa and helminths has recently been published by our group (24).

Although it is well established that *E. histolytica* induces host cell cytolysis directly through contact-dependent mechanisms (40), the involvement of amoebic-induced neutrophil products such as NETs in the pathology has not been evaluated. Moreover, our recent insights highlight the ability of amoebic trophozoite´s extracellular vesicles (membranous bodies carrying bioactive molecules and mediating intercellular communication) to modulate neutrophil effector responses, showing that amoeba can also affect neutrophils and tissues in a contact-independent manner (41).

In this study, we have explored the dark side of NETs by identifying the presence of NET-like structures in histological sections of hamster livers with amoebic liver abscess (ALA), assessing the cytotoxicity of purified amoebic-induced NETs on monolayers of colon and liver cells *in vitro*, and determining the NET components that mediate damage.

## 2 Materials and methods

### 2.1 *Entamoeba histolytica* culture

*E. histolytica* (strain HM1: IMSS) was cultured in TYI-S-33 medium supplemented with 15% heat-inactivated adult bovine serum (Microlab) and 3% NCTC-107 vitamins (Microlab) for 72 h at 37°C. The trophozoites were detached from the flask by ice chilling for 5 min and centrifuged at 1400 rpm for 5 min at 10°C. The cell pellet was resuspended in RPMI-1640 medium (Sigma) supplemented with 1% penicillin/streptomycin (Gibco) and maintained at 37°C until use.

### 2.2 Neutrophil isolation

Neutrophils were obtained from the peripheral blood of healthy volunteers according to the method of García-García et al. (42), using a Ficoll-Paque^®^ gradient (GE Healthcare) and hypertonic shock to lyse erythrocytes. Cells were resuspended in PBS pH 7.4, counted in a hemocytometer, and kept at 4°C until use. This study was conducted in accordance with the recommendations and approval of the Ethics Committee for Human Studies at the Institute of Biomedical Research, UNAM (ethical approval number: FMED/CI/RGG/013/01/2018). All subjects signed the informed consent prior to blood collection.

### 2.3 Induction, quantification, and isolation of NETs

For NET induction, neutrophils (5 x 10^5^) were centrifuged at 4000 rpm for 2 min and resuspended in 500 µl of RPMI-1640 medium (Sigma) supplemented with 5% fetal bovine serum (FBS, Gibco) and 500 nM SYTOX^®^ Green (Invitrogen). A 100 µl volume of the cell suspension (1 x 10^5^ neutrophils) was added to a 96-well plate, allowed to settle for 20 min at 37°C, and then stimulated with PMA (50 nM), A23187 (10 µM), or 1 x 10^3^, 2 x 10^3^, 5 x 10^3^ viable *E. histolytica* trophozoites (neutrophil-trophozoite ratios 100:1, 50:1, and 20:1, respectively). Fluorescence intensity was measured every 15 min from the bottom of the well during 4 h of incubation at 37°C in the multimodal plate reader Synergy HTX (Bio Tek) using 485 nm excitation and 528 nm emission filters.

NET purification was performed as described previously (43) with some modifications. After induction as mentioned above without SYTOX^®^ Green, the culture medium was carefully discarded to avoid disturbing the layer of NETs formed at the bottom of the well, which was then carefully washed once with cold PBS. Subsequently, 500 µl (800 µl for the NE and MPO enzymatic activity assay) of RPMI-1640 supplemented with 1% penicillin/streptomycin was added, and the adhered material (NETs) was mechanically and carefully pipetted to disperse it. The resulting dispersion from each treatment was centrifuged for 1 min at 2500 rpm, and the obtained supernatant (cell-free dispersed NETs, SnNET) was used for subsequent experiments. DNA and protein from SnNET were quantify using a UV spectrometer NanoDrop2000 (Thermo Fisher).

### 2.4 HCT 116 and Hep G2 cell lines

HCT 116 (CCL-247™) and Hep G2 (HB-8065™) cells were cultured in Petri dishes (Corning, 100 mm x 20 mm) in RPMI-1640 and OptiMEM^®^ media (Gibco) supplemented with 10% FBS, respectively, for 72 h at 37°C until reaching 90% confluency. The cells were detached using 0.05% EDTA-trypsin (Gibco), centrifuged at 1200 rpm for 5 min, and washed with sterile PBS. The cells were then centrifuged again under the same conditions, and the pellet was resuspended in the appropriate growth medium for cell reseeding.

### 2.5 Co-incubation assays of monolayers with amoebae and neutrophils

An amount of 5 x 10^3^ HCT 116 or Hep G2 cells were cultured in a 96-well plate for 72 h at 37°C until reaching 90% confluency. After this, 1 x 10^5^ neutrophils resuspended in 100 μl of RPMI-1640 or OptiMEM^®^ were deposited in the 72 h HCT-116 and Hep G2 cell cultures. Neutrophils were pelleted for 10 min at 37°C and stimulated with 5 x 10^3^ trophozoites for 0.5, 1 and 5 h at 37°C with 5% CO_2_ atmosphere. Cultures were fixed with 4% v/v formaldehyde for 15 min and washed once with PBS. Controls of neutrophils (1 x 10^5^) and amoebae (5 x 10^3^) cultured individually in cell monolayers were subjected to the same procedure. Samples were observed under an inverted microscope (Nikon) at 10X. Images obtained were processed with ImageJ software.

### 2.6 Monolayers viability assays

An amount of 5 x 10^3^ HCT 116 or Hep G2 cells were cultured in a 96-well plate for 72 h at 37°C until reaching 90% confluency. Afterwards, the growth medium was removed, and 100 µl of the total NET fraction obtained from neutrophil-amoeba co-cultures at 20:1, 50:1 and 100:1 ratio was added to each well (on average, 4 ± 0.6 µg, 2.5 ± 0.3 µg, and 1.9 ± 0.25 µg, respectively). In other experiments, the cell monolayers were incubated with SnNET or SnNET treated with inhibitors (see below) for 24 h at 37°C in 5% CO_2_.

The viability of HCT 116 cells was determined by removing SnNET from the culture and adding 100 µl of 3-[4,5-dimethylthiazol-2-yl]-2,5-diphenyltetrazolium bromide (MTT; 100 µg/ml in PBS) for 2 h at 37°C in the dark. Afterwards, the supernatant was discarded, and formazan salts were solubilized by adding 100 µl of a 0.01N HCl/10% SDS solution for 1 h at 37°C in the dark. DMSO (20%) was used as a death control. Absorbance was measured at 595 nm using the plate reader Thermo Scientific Multiskan FC.

The viability of Hep G2 cells was determined by removing SnNET from the culture and adding 50 µl of Alamar Blue^®^ reagent (Invitrogen) diluted 1:10 in PBS. Cells were then incubated at 37°C for 10 min in the dark, and absorbance was measured at 570 nm and 620 nm in a Synergy HTX.

### 2.7 NET inhibitors assay

SnNET (500 µl) were treated with sivelestat (ST, 10 µM), luminol (Lu, 50 µM), p-aminobenzohydrazide (iMPO, 40 µM), fluoromethylphenylsulfonide (PMSF, 0.1 mM), DNase I (3 U), and a combination of inhibitors and scavengers at the concentrations mentioned (Mix) for 30 min at 37°C. Afterwards, 100 µl of the treated SnNET were added to the monolayer cultures of HCT 116 or Hep G2 for 24 h and viability was determined as previously mentioned (the point describing the viability assays).

### 2.8 NE and MPO activity assay

For NE activity, 100 µl of SnNET were added to the chromogenic protease substrate (MeOSuc-AAPV-pNA; 500 µM; Santa Cruz Biotechnology) and shaken for 5 min at room temperature, followed by incubation for 1 h at 37°C in the dark. Absorbance was measured at 405 nm on the Synergy HTX.

For MPO activity, 100 µl of SnNET were added to luminol (800 µM) (Sigma), and immediately, 30% v/v hydrogen peroxide (Sigma; diluted 1:20,000) was added as a substrate to initiate the reaction. Luminescence was measured at the endpoint on the Synergy HTX.

### 2.9 ROS detection in Hep G2 cells

For ROS detection, a monolayer of 72 h Hep G2 cells was washed three times by adding and removing 100 µl of PBS and then 100 µl of ROS indicator in PBS (H_2_DCFDA; 50 µM) was added for 15 min at 37°C. The indicator was removed, cells were washed twice with PBS as indicated, and left in a volume of 100 µl of PBS. Fluorescence was read at the bottom of the well over a 100 min interval with an excitation and an emission filter 485/528 nm using the Synergy HTX.

For superoxide anion detection, a monolayer of 72 h Hep G2 cells was washed three times by adding and removing 100 µl of PBS and then 50 µl of the superoxide anion indicator nitro blue tetrazolium 0.05% in PBS (Sigma) was added for 2 h at 37°C in the dark. Then, 60 µl of KOH (4 M) in PBS was added without removing the indicator and cells were incubated for 10 min at room temperature with shaking. Afterward, 120 µl of DMSO was added to the same well volume and cells were incubated again for 20 min at room temperature with shaking. Absorbance was measured as the endpoint at 620 nm using the Synergy HTX.

For hydrogen peroxide detection, the Intracellular Hydrogen Peroxide Assay Kit (Sigma) was used according with manufacturer instructions. In brief, a monolayer of 72 h Hep G2 cells was washed three times by adding and removing 100 µl of PBS and then 100 µl of hydrogen peroxide sensor (0.4 µl of hydrogen peroxide sensor per 100 µl of hydrogen peroxide diluent) was added for 30 min at 37°C in the dark. Afterward, the supernatant was discarded, the monolayer was washed by adding and removing 100 µl of PBS, and left in a volume of 100 µl of PBS. For positive control, 100 µl of hydrogen peroxide diluted in PBS (100 µM) was added instead of PBS alone. Fluorescence was read at the bottom of the well with an excitation and emission filters of 485/528 nm in the Synergy HTX. For fluorescence microscopy of hydrogen peroxide, the same procedure was performed except that the hydrogen peroxide detector was allowed to act for 5 min at 37°C in the dark, and shortly thereafter, samples were visualized on an inverted fluorescence microscope Olympus IX71 (Nikon) using the emission and excitation filters of 480/520 nm.

### 2.10 NET visualization

For NET immunofluorescence, 2 x 10^5^ neutrophils were resuspended in 200 µl of RPMI-1640 supplemented with 5% FBS, seeded onto 12 mm diameter coverslides pre-treated with L-polylysine (Sigma) and allowed to settle for 20 min at room temperature. Neutrophils were stimulated with 1 x 10^4^ trophozoites, PMA (50 nM), or A23187 (10 µM) and incubated for 4 h at 37°C in a 5% CO_2_ atmosphere. Co-cultures were fixed with 4% v/v formaldehyde for 20 min and washed three times with PBS. Each coverslide was treated with 100 µl of 0.1% Triton X-100 (BioRad) in PBS for 5 min to permeabilize the cells and the detergent was washed off with cold PBS. Then, 100 µl of blocking solution (1% bovine serum albumin, 300 mM glycine, 0.1% Tween 20^®^ in PBS; pH 7.4) was added for 30 min at room temperature. The blocking solution was removed and 100 µl of anti-MPO antibody (mouse-made against human MPO; Abcam) diluted 1:100 in PBS-Tween 0.1%-BSA 1% was added and incubated for 1 h at room temperature. Afterward, the coverslides were washed three times with cold PBS and 100 µl of FITC-conjugated secondary anti-mouse antibody (goat-made against mouse; the antigen used was total mouse IgG; Sigma-Aldrich) diluted 1:100 in PBS-Tween 0.1%-BSA 1% was added and incubated for 1 h at room temperature in the dark. The preparations were washed once with PBS and 100 µl of DAPI (5 µg/ml) was added for 15 min in the dark. The coverslides were washed once more with PBS and mounted with FluoroShield^®^ (Sigma). Samples were observed under an Olympus BX51 fluorescence microscope with excitation filters of 350/480 nm and emission filters of 460/520 nm. The obtained images were processed using ImageJ software.

For NET fluorescence in HCT 116 and Hep G2 cell monolayers, 1 x 10^5^ neutrophils resuspended in 100 µl of RPMI-1640 or OptiMEM^®^ were deposited in the cultures of 72 h HCT 116 and Hep G2 cells. Neutrophils were settled for 10 min at 37°C and then stimulated with 5 x 10^3^ trophozoites for 4 h at 37°C in 5% CO_2_ atmosphere. The triple cell culture was fixed with 4% v/v formaldehyde for 15 min and washed once with PBS. Following this, 100 µl of DAPI (5 µg/ml) were added to the cultures for 15 min in the dark and the samples were visualized on an inverted fluorescence microscope Olympus IX71 using an excitation filter of 350 nm and an emission filter of 460 nm.

### 2.11 ALA induction

An *E. histolytica* trophozoite culture of 72 h was centrifuged at 1400 rpm for 5 min. The supernatant was removed, and the cells were resuspended in PBS to achieve a concentration of 10^6^ trophozoites in 100 µl. Male golden hamsters (*Mesocricetus auratus*) of 6 weeks old and approximately 100 g weight were anesthetized with sodium pentobarbital (Pet’s Pharma), the peritoneal cavity opened by surgical laparotomy, and the portal vein exposed by removing the intestines from the abdominal cavity. A volume of 100 µl of parasites (1 x 10^6^ trophozoites) was directly inoculated in the portal vein bloodstream, the site of inoculation immediately occluded with gel foam pads, the intestines returned to the peritoneum and the abdomen sutured using vyclil 4-0. On the seventh day, the hamsters were euthanized using excess of anesthesia. The livers were excised and abscesses pieces fixed in 4% paraformaldehyde and stored in 30% sucrose for histology. Tissue sections of 5 µm were obtained in a microtome and Periodic Acid-Schiff (PAS) stained for microscopy. For NET visualization in liver tissue, immunofluorescence was performed as described above. In order to observe chromatin decondensation, immunofluorescence for NE was performed using a Rhodamine-conjugated secondary anti-mouse antibody (Pierce) followed by the NUCLAER-ID^®^ Green Chromatin Condensation Detection Kit (Enzo) according to manufacturer instructions. Samples were observed under an Olympus BX51 fluorescence microscope with excitation filters of 350/480 nm and emission filters of 460/520 nm. The obtained images were processed using ImageJ software. The protocol was approved by the Institutional Animal Care and Use Committee of the Faculty of Medicine, UNAM with identification number CICUAL 5427 (42).

### 2.12 Statistical analysis

The obtained data were analyzed using one-way ANOVA with Tuckey *post-hoc* test or Kruskall-Wallis with Dunn *post-hoc* test using GraphPad software. Graphs represent average ± standard deviation.

## 3 Results

### 3.1 NETs are associated to necrosis in ALA

**Figure 1:**
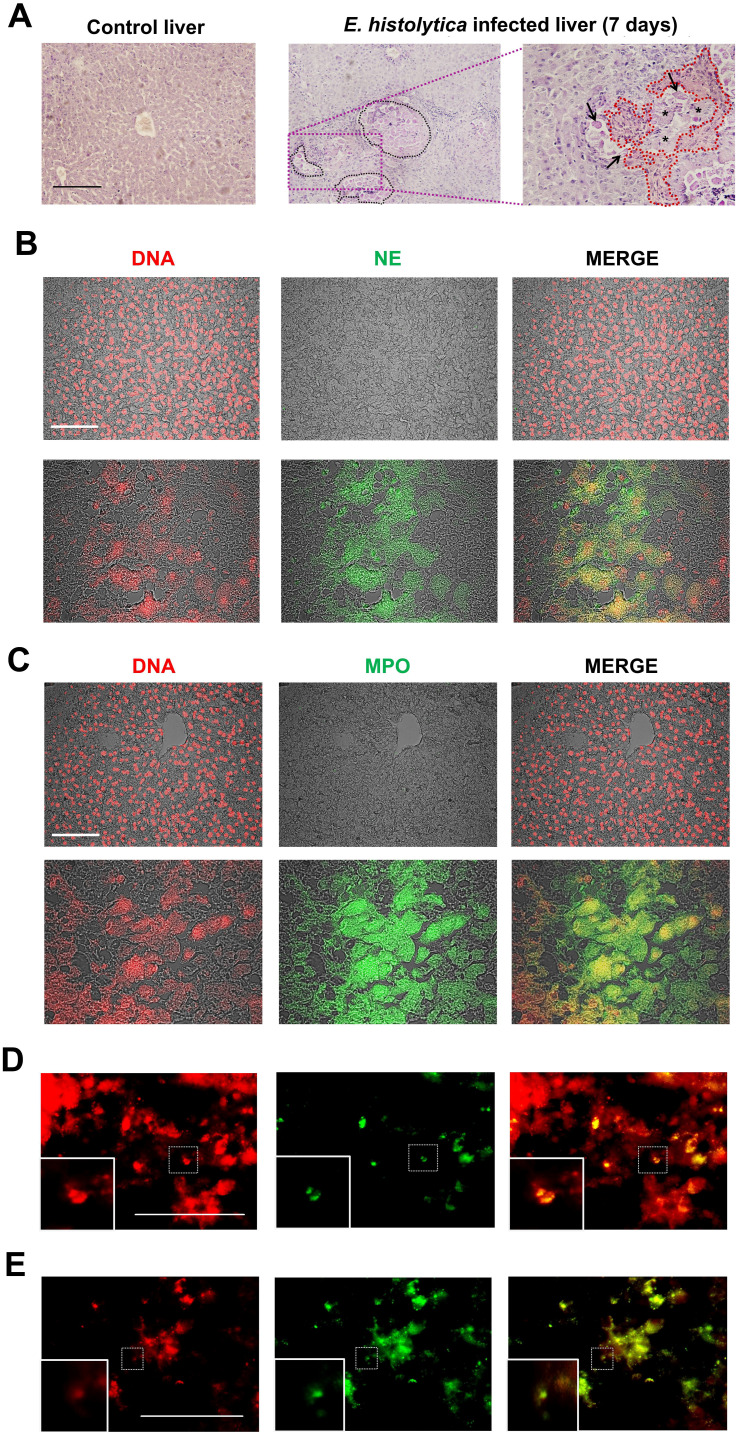
Amoebic liver abscesses contain NET-like structures. **(A)** Histological sections of a healthy liver and a liver infected with *E. histolytica* trophozoites after 7 days of infection stained with PAS. Black dotted lines in demarcate microabscesses filled with amoebae and necrotic tissue foci (asterisks), accompanied with a massive infiltration of immune cells surrounding these areas. A 40x magnification (purple dotted lines) also shows trophozoites (black arrows), inflammatory infiltrate (red dotted lines) and adjacent necrotic areas (asterisk). **(B, C)** Immunofluorescence images of amoebic liver microabscesses revealing the presence of neutrophil elastase (NE) and myeloperoxidase (MPO) (both in green), respectively, and their co-localization with DNA (in red), as seen in the merge boxes (yellow and orange). Areas of co-localization are considered as NETs. Chromatin decondensation was evidenced using the NUCLEAR-ID^®^ Green Chromatin Condensation Detection Kit, showing neutrophils in early NETosis (**D** condensed nucleus colocalizing with NE) or in late NETosis (**E** decondensed nucleus colocalizing with NE). All scale bars: 100 μm. SnPMN: cell-free supernatant obtained from neutrophils culture only.

We analyzed the histology of *ex vivo* liver tissues 7 days after infection with *E. histolytica* in order to find evidence of NET formation associated with tissue damage. As expected, the infected tissue showed the development of micro-abscesses containing necrotic areas surrounded by localized amoebae, as well as changes in the normal architecture of the hepatocyte cords compared to healthy tissue (**Figure 1A**). Additionally, the inflammatory activity was notably intense due to the abundance of immune cells in the hepatic sinusoids as well as at the periphery of the abscesses where the amoebae were located. When the tissue sections were tested for the common NETs components (DNA, NE, and MPO) by using immunofluorescence, no evidence of NETs was observed in healthy tissue (**Figures 1B, C**, top images). In contrast, in tissues with ALA, extensive necrotic areas were observed and NET markers (NE and MPO) were identified colocalizing with extensive regions of extracellular DNA resembling cloud-like structures, located in proximity to the damaged hepatocytes (**Figures 1B, C** lower images). Noteworthy, neutrophils in early and late stages of NETosis, as determined by the presence of condensed nuclei colocalized with NE (**Figure 1D**) or decondensed nuclei with colocalization of NE (**Figure 1E**), respectively, were observed near the NETs.

### 3.2 *E. histolytica*-induced NETs interact with colon and liver cells *in vitro*

We first confirmed that *E. histolytica* trophozoites were capable of inducing NETosis *in vitro*. **Supplementary Figure 1** shows that amoebae induced the release of cloudy NETs which contrast with the fibrillar NETs induced by positive controls PMA or A23187. MPO immuno-staining confirmed that the observed structures were NETs, but also showed that the distribution of MPO was more heterogeneous in amoeba-induced NETs compared to PMA or A23187-induced NETs.

**Figure 2:**
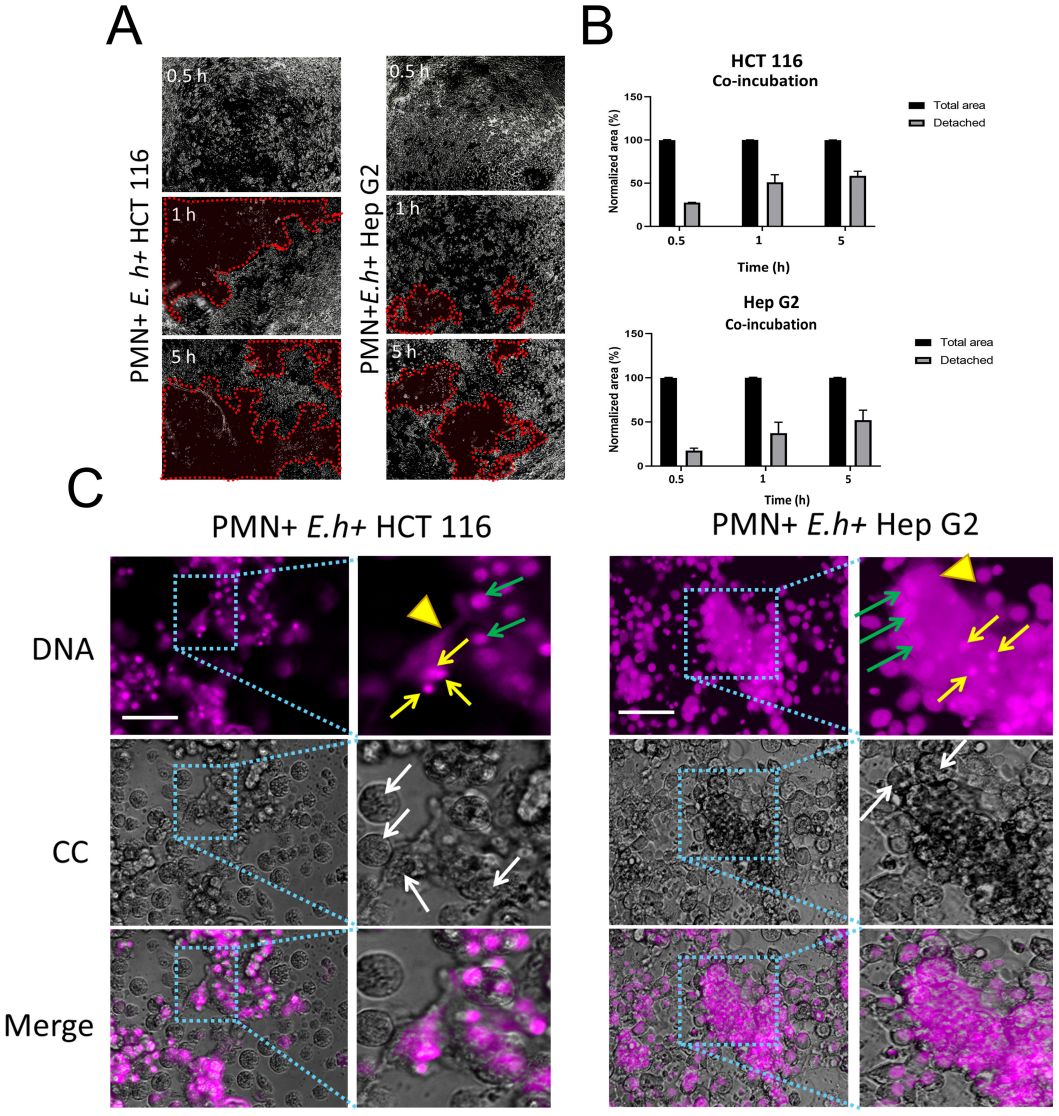
NETs induction by *E*. *histolytica* trophozoites and their effect on colon and liver cell monolayers. **(A)** Neutrophils (1 x 10^4^) and trophozoites (5 x 10^3^) were co-cultured on confluent (>90%) monolayers of HCT 116 or Hep G2 cells (72 h of grown) for 0.5, 1, and 5 h. Red dotted lines highlight areas of cell detachment. Samples were fixed, and images were captured using an inverted brightfield microscope (Nikon) at 5x magnification. **(B)** Quantification of detached area. **(C)** Neutrophils (1 x 10^4^) and trophozoites (5 x 10^3^) were cultured on a confluent (>90%) monolayer of HCT 116 or Hep G2 cells for 5 h. Samples were fixed, and DNA was stained with DAPI. Light blue boxes highlight areas of interaction between NETs, amoebae, and cell monolayer. Yellow arrows indicate neutrophils, arrowheads indicate NET-like structures, white arrows indicate trophozoites and, green arrow indicate nuclei of HCT 116 or Hep G2 cells. Images were obtained using an inverted fluorescence microscope (Nikon) at 20x magnification. For **(A, C)**: scale bars represent 100 μm. E. h, *E*. *histolytica* trophozoites.

Posteriorly, we evaluate the effect of *E. histolytica* induced NETosis occurring in the presence of hepatic or colonic cell monolayers. When HCT 116 (colonic) and Hep G2 (hepatic) cell monolayers were exposed to neutrophils or amoebae separately, we did not observe any effect of neutrophils on HCT 116 and only a slight detachment of Hep G2 after 5 h of incubation. Amoebae, on the other hand, detached part of the monolayers of both cell lines by a maximum of 25% at 5 h (**Supplementary Figure 2**). In contrast, when neutrophils and amoebic trophozoites were co-cultured simultaneously over HCT 116 or Hep G2 cell lines, we observed extensive detachment in the monolayer of both cell types in a time-dependent manner, suggesting that co-incubation of the three cell types has increased cytotoxic effect (**Figure 2A**). When the detached area was quantified, the effect was most notable at early times (0.5 and 1 h) on HCT 116 cells, but subsequently both cell types showed a detachment of around 50% at 5 h. (**Figure 2B**). Cells detachment and damage was associated with NET formation, which were located surrounding the trophozoites and concentrated in the detachment areas of both colon and liver cells (**Figure 2C**).

### 3.3 *E. histolytica*-induced NETs mediate cytotoxicity on colonic and hepatic cells lines

**Figure 3:**
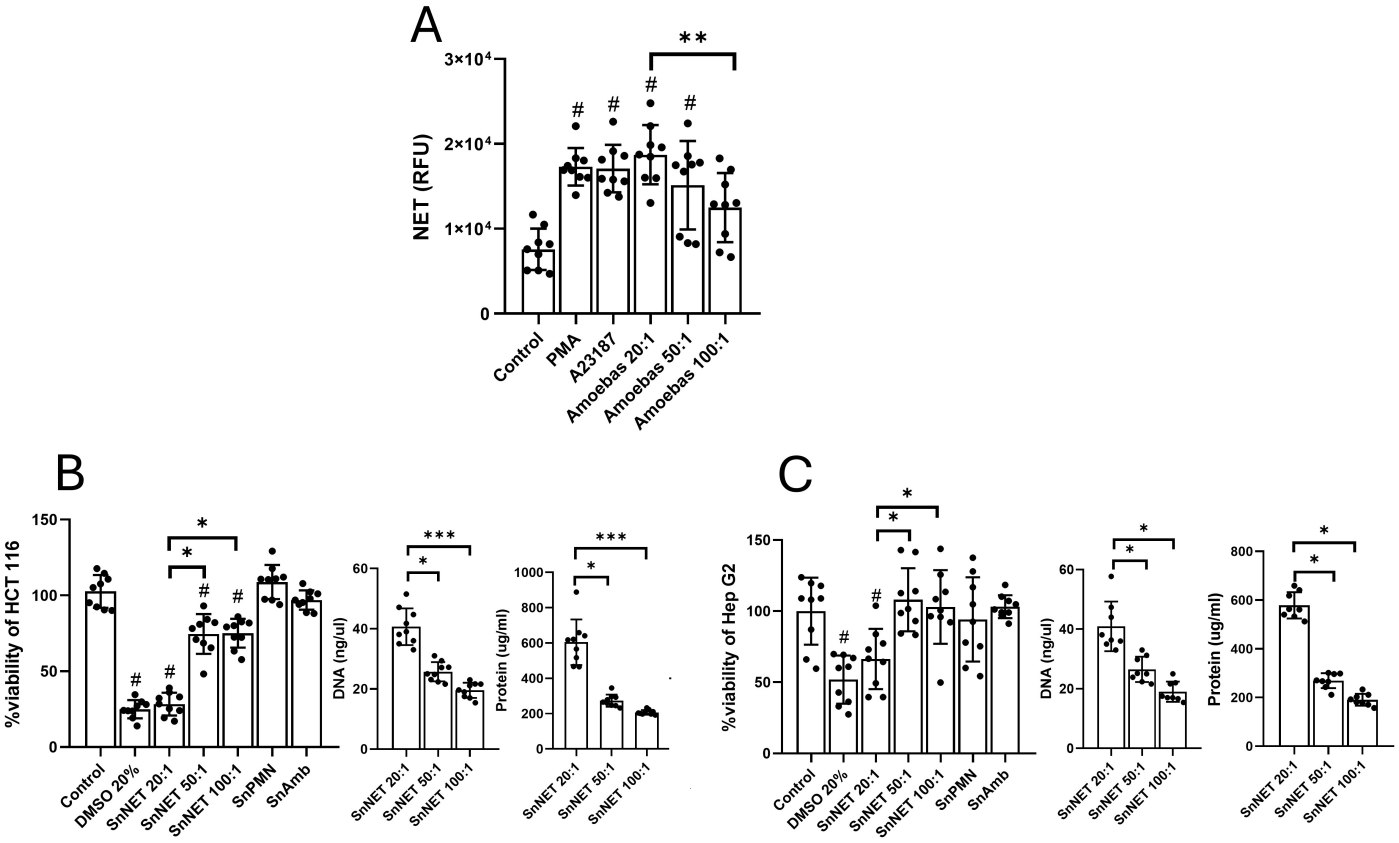
NETs induced by *E*. *histolytica* trophozoites affect the viability of colon and liver cell monolayers *in vitro*. **(A)** Human neutrophils (1 x 10^5^) were stimulated with viable *E*. *histolytica trophozoites* (5 x 10^3^, 2 x 10^3^, and 1 x 10^3^) at neutrophil:amoeba ratios of 20:1, 50:1, and 100:1, respectively, and extracellular DNA was quantified in relative fluorescence units (RFU) using SYTOX Green^®^ (500 nM). PMA (50 nM) and A23187 (10 μM) were used as positive controls. NETs were purified from the previous samples, and the cell-free supernatant (SnNET) was added to confluent monolayers of HCT 116 cells **(B)** or Hep G2 cells **(C)** for 24 h. After this time, cell viability was assessed using the MTT assay. The amount of DNA and protein in the SnNET was quantified using a UV spectrometer (NanoDrop 2000) at 260 nm and 280 nm, respectively, before being added to cells. DMSO (20%) was used as a positive control for cytotoxicity and cell lines death. Data are shown as means from three independent experiments, each performed in triplicate. (#) indicates a statistical difference compared to the control (*p* < 0.05). *(*p* < 0.05), **(*p* < 0.01), and ***(*p* < 0.001). SnPMN, cell-free supernatant obtained from neutrophils culture only; SnAmb, cell-free supernatant obtained from amoebae culture only.

To determine if NETs participate in the monolayer damage during the co-cultures, we purified NETs induced by *E. histolytica* (SnNET) and their effect on the cell lines was evaluated. Initially, we measured the release of NETs at different neutrophil:amoeba ratios (20:1, 50:1, and 100:1) observing that trophozoites induced NET release in a dose-dependent manner, with the 20:1 ratio inducing the highest amount, statistically equivalent to the amount of NETs released with the positive controls PMA and A23187 (**Figure 3A**). Posteriorly, the SnNET fractions were added to HCT 116, and Hep G2 cell monolayers and their viability was assessed at 24 h (**Figures 3B, C**, respectively). To correlate the effect of the NETs with the concentration of their components, the DNA and protein concentration of the three neutrophil-amoeba co-culture ratios was determined. The results show that SnNET induced dell death in HCT 116 in a dose-dependent manner, with SnNET 20:1 (about 4 µg DNA/100 µl) exhibiting the highest activity, killing around 75% of the cells compared to untreated control (**Figure 3B**). This result was similar to the one obtained with the death control (20% DMSO) (**Figure 3B**; *p* < 0.01). On the other hand, SnNET 50:1 and SnNET 100:1 (about 2.5 and 1.9 µg DNA/100 µl) killed 25 to 30% of the cells compared to the untreated control (*p* < 0.05), with no statistical difference between them. The number of dead cells correlated with the DNA and protein concentration in each sample (**Figure 3B**). In the case of Hep G2 cell line, SnNET also decreased the cell viability in a dose-dependent manner, but the effect was less marked than with HCT 116 cells (**Figure 3C**). Thus, SnNET 20:1 killed 35% of the cells when compared to the untreated control (*p* < 0.05), while SnNET 50:1 and SnNET 100:1 did not affect the viability of the monolayer. Cell death also correlated with the concentration of DNA and protein present in SnNET 20:1 (**Figure 3C**). It is important to mention that fractions from individual cultures of neutrophils and amoebae that underwent the same purification procedure showed no effect on the viability of HCT 116 and Hep G2 cells (**Figures 3B, C**, SnPMN and SnAmb, respectively).

### 3.4 The cytotoxicity of amoeba-induced NETs on HCT 116 cells is mediated by serine proteases, excluding NE

To explore the contributions of the components of *E. histolytica*-induced NETs on the colon and liver cell monolayers cytotoxicity, we used a battery of inhibitors and scavengers.

**Figure 4:**
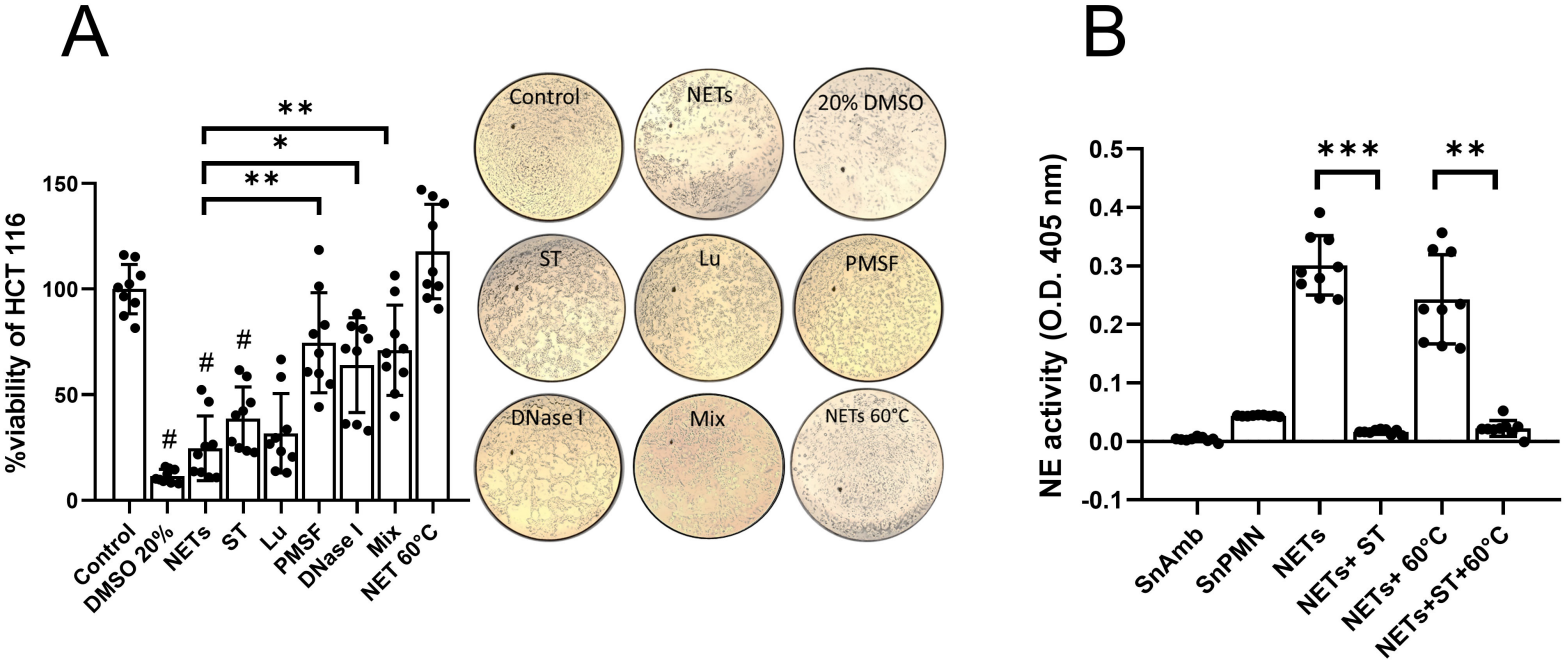
Effect of inhibitors and degraders of NETs components on the cytotoxic effect of NETs in HCT 116 cells. **(A)** Confluent HCT 116 monolayers were treated for 24 h with purified NETs (SnNET 20:1) or with NETs that were pretreated with sivelestat (ST; 10 µM), luminol (Lu; 50 µM), fluoromethylphenylsulfonyl fluoride (PMSF; 0.1 mM), DNase I (3 U), a combination of all treatments (Mix), or by heating to 60°C. Cell viability was determined using the MTT assay. Images of the treated monolayers after 24 h at 10x magnification are shown (middle). **(B)** NE activity in NETs (SnNET 20:1) without treatment or heating to 60°C, in the absence or presence of ST (10 µM). Data are presented from three independent experiments, each performed in triplicate. (#) indicates a statistical difference compared to the control (*p* < 0.05). *(*p* < 0.05), **(*p* < 0.01), and ***(*p* < 0.001). SnPMN: cell-free supernatant obtained from neutrophils culture only.

Since SnNET 20:1 (hereafter referred to as NETs) induced the highest percentage of cell death, we proceeded to pretreat NETs with sivelestat (ST), a NE inhibitor; luminol (Lu), a scavenger of MPO-derived ROS; fluoromethylphenylsulfonyl (PMSF), a general inhibitor of serine proteases; DNase I to degrade the DNA scaffold; and a combination of all compounds (Mix). The results revealed that pretreatment of the NETs with PMSF and DNase I significantly attenuated the death percentage of HCT 116 cells compared to untreated NETs, improving viability from 24.17 ± 15% to 71.9 ± 23% and 64.6 ± 21%, respectively (**Figure 4A**). NETs pretreatment with Lu and ST also improved the viability of HCT 116 cells, but no significant differences were observed compared to untreated NETs. Interestingly, pretreatment of the NETs with the mix did not show an additive effect on improving the viability of HCT 116 cells, being statistically equivalent to the effect of PMSF and DNase I alone (**Figure 4A**). These results were confirmed visualizing the cell monolayers after each treatment observing the cell detachment. To differentiate the contribution of DNA and serine proteases in the cytotoxicity of colon cells, we performed a thermal pretreatment of NETs, finding that they completely lost their cytotoxic effect, indicating that the cytotoxic component is thermolabile (probably serine proteases). Although NE activity was detected in NETs and decreased, as expected, in the presence of ST (**Figure 4B**), it did not decrease in heat-treated NETs (except in the presence of ST again; **Figure 4B**), corroborating that the cytotoxic activity of the NETs on HCT 116 colon cells is independent of NE, but dependent on the activity of other serine proteases.

### 3.5 The cytotoxicity of amoeba-induced NETs on Hep G2 cells is mediated by MPO activity

**Figure 5:**
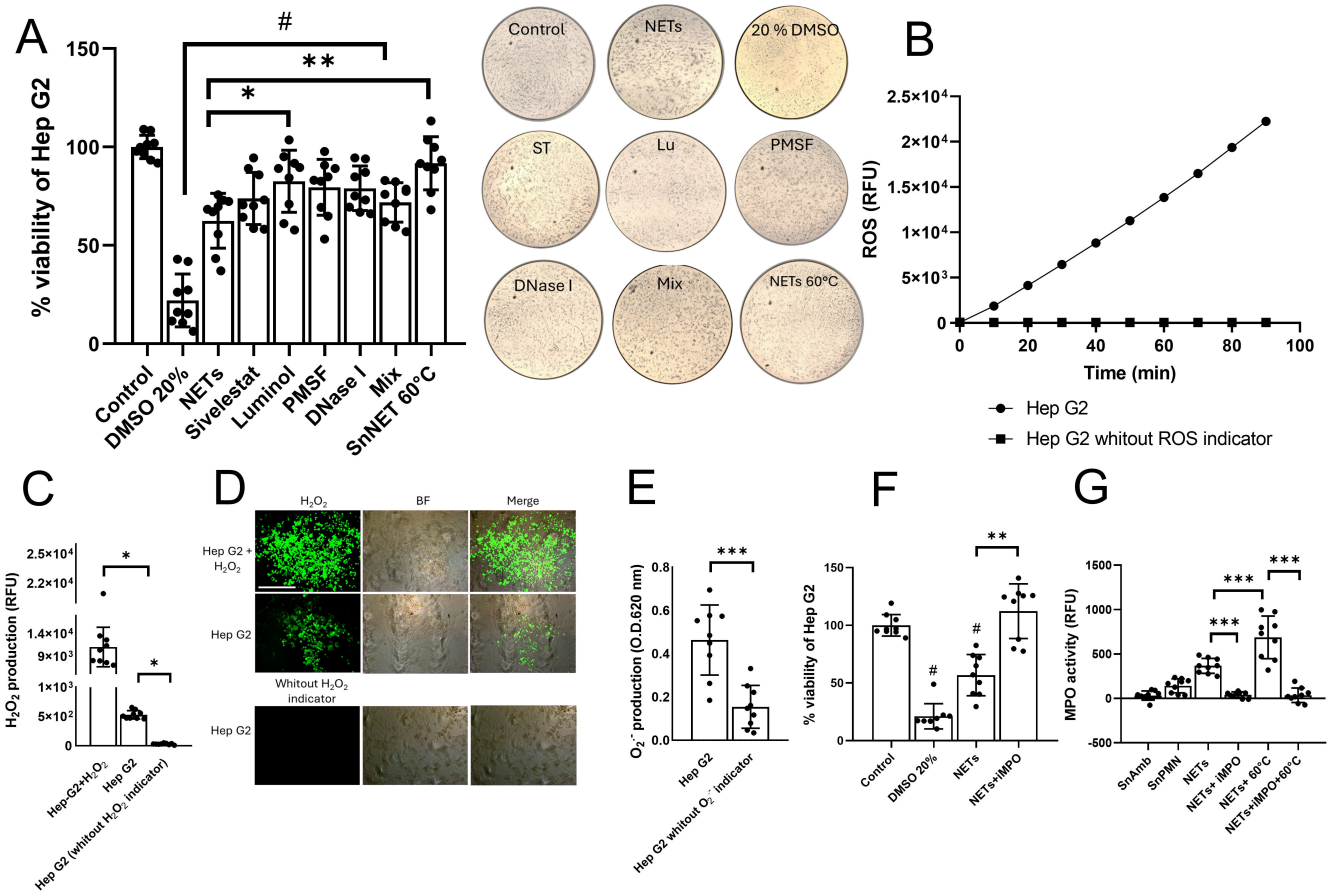
Effect of inhibitors and degraders of NETs components on the cytotoxic effect of NETs in Hep G2 cells. **(A)** NETs from neutrophils (3 x 10^6^) incubated with viable trophozoites (1.5 x 10^5^; SnNET 20:1) in the presence or absence of sivelestat (ST; 10 µM), luminol (Lu; 50 µM), fluoromethylphenylsulfonyl fluoride (PMSF; 0.1 mM), DNase I (3 U), and a combination of all NET inhibitors (Mix) was added to a confluent monolayer of Hep G2 cells for 24 h. Cell viability was assessed using the MTT assay. Images of the treated monolayers after 24 h at 10x magnification are shown (middle). **(B)** A monolayer of Hep G2 cells was pre-treated with the ROS indicator H_2_DCFDA for 10 min to measure ROS production over a 90-min interval. **(C)** A monolayer of Hep G2 cells was pretreated with a hydrogen peroxide detector for 30 min to measure hydrogen peroxide production using excitation and emission filters of 485/40 nm and 528/20 nm, respectively **(D)** or analyzed by fluorescence microscopy using an inverted fluorescence microscope (Nikon) at 10x magnification scale bar represents 100 μm. **(E)** A monolayer of Hep G2 cells was pretreated with a superoxide anion indicator to measure superoxide anion production. Absorbance was measured at 620 nm. **(F)** NETs from neutrophils (3 x 10^6^) incubated with viable trophozoites (1.5 x 10^5^; SnNET 20:1) in the presence or absence of iMPO (p-aminobenzohydrazide, 40 µM) was added to a monolayer of Hep G2 cells for 24 h. Cell viability was determined using the MTT assay. **(G)** MPO activity was measured in NETs from neutrophils (3 x 10^6^) incubated with viable trophozoites (1.5 x 10*^5^*; SnNET 20:1) in the absence or presence of iMPO (40 µM) and after heating to 60°C in the absence or presence of iMPO. For **(A, F)** DMSO (20%) was used as a positive control for cytotoxicity and Hep G2 line cell death. (#) indicates a statistical difference compared to the control (*p* < 0.05). *(*p* < 0.05), **(*p* < 0.01), and ***(*p* < 0.001). SnAmb, cell-free supernatant obtained from amoebae culture only. SnPMN: cell-free supernatant obtained from neutrophils culture only.

In the case of Hep G2 cells, we observed that pretreatment of the NETs with ST, PMSF, and DNase I slightly reduced their cytotoxic effect, but not to a statistically significant extent (**Figure 5A**). Only the pretreatment with luminol (Lu) statistically improved the viability of Hep G2 cells compared to the cytotoxicity control with untreated NETs (from 62.45 ± 13% to 82.56 ± 15% survival, *p* < 0.05). Similar to what was observed with HCT 116 cells, we did not find that the mix of inhibitors and eliminators attenuated the cytotoxic effect of the NETs beyond what was observed with Lu, suggesting that MPO activity is responsible for the effect. Accordingly, heat denaturation pretreatment of the NETs abolished their cytotoxic effect on Hep G2 cells (**Figure 5A**). These results were also confirmed visualizing the cell monolayers after each treatment observing the cell detachment. Since Lu is a scavenger of the enzymatic product of MPO, hypochlorous acid (HClO), which is produced from the reaction between chloride ions in the medium and hydrogen peroxide, we proceeded to determine if Hep G2 cells could be the source of hydrogen peroxide for the formation of HClO. First, we evaluated if Hep G2 cells produce general ROS using the ROS indicator H_2_DCFDA. **Figure 5B** shows that, over a period of 90 min, Hep G2 cells produce ROS that accumulate over time. Using a specific hydrogen peroxide indicator, we demonstrated that Hep G2 cells produce hydrogen peroxide among their ROS (**Figure 5C**) and that it can be detected inside the cells by fluorescence (**Figure 5D**). Likewise, we measured the production of superoxide anion, a precursor of hydrogen peroxide, and detected that Hep G2 cells also generate this ROS type (**Figure 5E**).

To corroborate that the cytotoxic effect of the NETs on Hep G2 cells was due to MPO activity, we also evaluated the effect of a specific MPO inhibitor (iMPO; p-aminobenzohydrazide). The pretreatment of the NETs with iMPO prevented the death of Hep G2 cells, thus being a better treatment than Lu (**Figure 5F**). Additionally, we measured the enzymatic activity of MPO in the NETs and detected that MPO is indeed an enzyme that is catalytically active in the SnNET fraction, and its activity is statistically reduced with iMPO (**Figure 5G**). Curiously, heat denaturation pretreatment appears to have increased MPO activity, which was abolished in the presence of iMPO (**Figure 5G**).

## 4 Discussion

Early studies reported that *E. histolytica* trophozoites directly trigger host cell damage *in vitro* (44–46) through mechanical processes involving phagocytosis (46) and trogocytosis (47), as well as through the secretion of virulence factors including host cell adhesion molecules (40, 48, 49), mucin-2 degrading glycosidases (50), cysteine proteases (51, 52) and pore-forming peptides (44). However, in *in vivo* infection models, such as liver abscesses in hamsters, the extensive liver tissue damage for the small number of amoebae observed in histological sections has always been striking. Based on studies by our group and others, it is now discussed whether the host cellular immune response, and in particular neutrophils which are the most abundant cells, can contribute to the damage associated with invasive amebiasis (7, 9). Here, we present evidence of NETs formation in amoebic liver abscesses (ALA) and their association with areas of necrosis in liver tissue. Next, as the core of this work, we show for the first time that *E. histolytica*-induced NETs cause damage to colon and liver cells monolayers in *in vitro* cultures at early times post-exposure. Furthermore, we demonstrate that their cytotoxic activity is mainly mediated by the activity of serine proteases on colon cells and MPO on liver cells, indicating that the NET components may exert cytotoxicity differentially depending on the tissue type.

Since *E. histolytica* rapidly and intensely induces NETs release and their role in defense remains unclear (13, 18), our hypothesis was that NETs may play an important role in the cell damage associated with amebiasis due to their pathological properties described in other diseases (29, 32, 33, 53). To support this possibility, we used immunofluorescence to detect three characteristic components of NETs: DNA, MPO, and NE, in histological sections of ALA. DNA staining revealed the dissolution of nuclei in the liver tissue and extensive regions of DNA in cloud-like formations adjacent to damaged areas that colocalized with the NET markers. Additionally, neutrophils in early stages of NETosis were observed, showing decondensed nuclei in the cytoplasm with co-localization of MPO and NE. In agreement, another study reported in a mouse model of ALA a large number of apoptotic hepatocytes adjacent to neutrophil infiltrate without showing close contact with the amoebae (6). The presence of NETs *ex vivo* in bacterial (54–57), fungal (58), viral (59, 60), and parasitic (28, 61, 62) infectious diseases, as well as in autoimmune diseases (63–66), has also been reported. The relevance of NETs in liver tissue damage has been reported in a murine model of sepsis induced by *Staphylococcus aureus*, where NETs induced by the bacteria in hepatic sinusoids contribute to tissue destruction, but not when the mice are treated with DNase (56). Similar studies are currently being carried out in our laboratory.

Once demonstrated the presence of NETs in ALA, to simulate the encounter scenario of amoebae with neutrophils in the tissue, we first exposed monolayers of colon (HCT 116) and liver (Hep G2) cells, *E. histolytica* target cells, to a co-culture of amoebae:neutrophils at different ratios. In contrast to neutrophils stimulated with PMA and A23187 that formed NETs with an extended morphology, consistent with previous classifications (67), the NETs formed in response to *E. histolytica* exhibited a diffuse, cloud-like morphology (67) with a heterogeneous distribution of MPO, dispersed over the extracellular DNA, as previously described (68). These neutrophil-amoeba co-culture NETs resembled the NET-like structures we identified in histological sections of ALA. Regarding the monolayers, we quickly observed signs of damage, evidenced by the extensive detachment of colon cells after 1 h and liver cells after 5 h. Although amoebae and neutrophils alone were able to detach part of the monolayers at 5 h, twice as much detachment of the monolayers was observed with co-incubation and was associated with the appearance of NETs. This result is in agreement with an old study by Salata and Ravdin showing the effect of the interaction of human neutrophils and *E. histolytica* on hepatocyte monolayers (Chang cells) over 3 h incubation (69). They did not detect changes in the cell monolayer after incubation with neutrophils alone but observed approximately 35% destruction of the monolayer after incubation with amoebae. However, when neutrophils and amoebae were incubated simultaneously, the percentage of hepatocyte monolayer destruction doubled compared to the effect observed with amoebae alone. They concluded that monolayer destruction was due to neutrophil lysis and not to potential molecules released by lysed amoebae, as the trophozoites remained 100% viable during the co-incubation period (69). Although Salata and Ravdin were unaware of the existence of NETs, which would explain the lysis of neutrophils, they also stimulated neutrophils with A23187 to evaluate the effect of neutrophil degranulation on hepatocyte monolayer destruction after 1 h. Under their experimental conditions (A23187 10 µM and 1 h of incubation), the slight increase in monolayer destruction they observed could also be attributed to NET formation induced by the calcium ionophore (69).

To demonstrate that amoeba-induced NETs have cytotoxic potential, we purified them from neutrophil-amoeba co-cultures at different ratios (SnNET 20:1, SnNET 50:1, and SnNET 100:1) and added to colon and liver cell monolayers to assess their viability. In both cell lines, we observed a cytotoxic effect of NETs that was dependent on the concentration of DNA and protein, but notably, the effect was much more evident in colon HCT 116 than in liver Hep G2 cells. The high susceptibility of colon cells to NETs has also been reported in the Caco-2 cell line, used in studies of inflammatory bowel disease (70, 71). In these studies, NETs induced ~40% (70) and ~55% (71) death in colon cells, attributed to an increase in Caco-2 cell permeability, which is lower than the 75% death rate we report here. Cytotoxicity of NETs on liver sinusoidal endothelial cells (LSEC) has also been reported (72). However, the authors concluded that LSEC damage was primarily associated with the activation of the coagulation cascade and macrophages. Obviously, the possible contribution of these mechanisms to the damage associated with NETs during amoebic invasion of the intestine or liver cannot be ruled out. It should be noted that PMA-induced NETs and lower NET DNA concentrations were used in those studies, suggesting that, on the one hand, all NETs could have cytotoxic potential, but on the other hand, that the degree of cytotoxicity could vary depending on the stimulus that induces them. Although it appears that liver cells are less susceptible than intestinal cells to the cytotoxic effects of amoeba-derived NETs, it is important to note that the experimental conditions in these studies differ significantly from those used in our study, making a direct comparison challenging.

NETs, often described as the spider webs of immunity, are structured by nuclear (73) and/or mitochondrial (74) DNA fibers, together with nuclear, granular, and cytoplasmic proteins that confer their antimicrobial (15) and mechanical properties (75). Proteomic studies of PMA-induced NETs have identified around 330 proteins, more than half of which are associated with inflammatory processes (15, 76, 77). Although the composition of NETs varies depending on the stimulus (76), these structures mainly consist of nuclear DNA, histones, neutrophil elastase (NE), myeloperoxidase (MPO), and antimicrobial peptides. To determine which components of *E. histolytica*-induced NETs caused the death of colon and liver cells, we pretreated the purified NETs with a series of inhibitors and scavengers of DNA, serine proteases, and MPO, before applying them to the monolayers. Our findings showed that NETs treated with the serine protease inhibitor PMSF and DNase I had their cytotoxic capacity reduced, but not significantly when NE inhibitor ST and the scavenger of MPO enzymatic product Lu were used, even though these enzymes remained catalytically active. It is noteworthy that while ST did not significantly reduce the death of HCT 116 cells, other studies have reported the cytotoxic potential of NE associated with NETs on epithelial cells (78–80). Therefore, the fact that PMSF significantly reduced the cytotoxic capacity of NETs induced by amoebae and that ST had a tendency to reduce it, suggests that NE, being a serine protease, may participate in the effect together with other neutrophil serine proteases such as cathepsin G, proteinase 3 and azurocidin. More detailed studies with specific inhibitors of the other serine proteases are required to demonstrate the contribution of each to the cytotoxicity of amoeba-derived NETs. On the other hand, we found that the cytotoxic activity of NETs was completely abrogated after heating, supporting the notion that pathologic properties of NETs are related to the protein fraction, and that proteins such as NE may be anchored to a DNA scaffold to enhance their toxic concentration. This could explain why although NE remained active after heating, the fraction lost its activity because the proteins dissociate from the DNA. Furthermore, it is important to mention that treatment of NETs with the mixture of inhibitors and scavengers did not completely prevent the death of cell monolayers, suggesting that there are other components of NETs, not analyzed here, that also contribute to NET-mediated cell damage. Such is the case of histones, abundant components of NETs that have shown cytotoxicity on epithelial cells (81–83), including colon cells (70). Further studies are required to determine the role of histones in amoebae-NET-mediated cytotoxicity.

As mentioned above, Hep G2 cells were less susceptible to amoeba-derived NETs, and only the pretreatment with Lu slightly improved hepatocyte viability. Since Lu is a specific scavenger of the MPO product HClO (84), which is generated from the reaction between halide ions in the medium and hydrogen peroxide (85), we hypothesized that Hep G2 cells produce their own hydrogen peroxide. As expected, we detected ROS, such as superoxide anion and hydrogen peroxide, within Hep G2 cells over time. The sources of ROS in hepatocytes are well characterized (86). NOX (87), mitochondria (88), peroxisomes (89), cytochrome P450 (90), and xanthine oxidase (91) in liver cells produce superoxide anion, which spontaneously dismutates or is catalyzed by superoxide dismutase to hydrogen peroxide (92). Therefore, under our experimental conditions, MPO from the NETs likely catalyzed the formation of its product from the hydrogen peroxide produced by Hep G2 cells, generating HClO as a mediator of damage to the Hep G2 cells themselves. To corroborate this, we also treated NETs with a specific MPO inhibitor (iMPO) showing that it completely abrogated their cytotoxicity on Hep G2 cells, even better than the Lu treatment. Interestingly, as with NE activity mentioned above, heating of NETs did not abolish MPO activity, but lost its ability to kill Hep G2 cells. This observation supports our proposal that heating of NETs may cause proteins such as NE and MPO to dissociate from DNA, becoming diluted in the supernatant and losing their ability to cause cell damage. This points to a very important role of NET DNA as a scaffold that supports and concentrates proteins to exert their effect on the target trapped by NETs, as it has been proposed (75, 93). Our findings also indicate that hydrogen peroxide produced by hepatocytes themselves can be exploited by the oxidative mechanisms of NETs to trigger damage during invasive amebiasis.

In conclusion, our study provides novel evidence regarding the pathological impact of NETs in amebiasis, specifically in the damage to colon and liver cells *in vitro*. The identification of NETs in histological sections of ALA in hamsters, along with their cytotoxic effect in *in vitro* models, suggests that these structures play a key role in the pathogenesis of the disease, probably through components such as serine proteases and MPO. Moreover, our findings suggest, beyond the limitations of *in vitro* cell models, that NET components might exert differential cytotoxicity depending on the tissue type. This work not only contributes to the understanding of the mechanisms underlying amoebic pathology, but also underlines the need to further explore the role of other NET components and their potential interaction with *E. histolytica*-derived EVs. This opens new perspectives for the development of therapeutic strategies aimed at mitigating tissue damage in this disease.

## Data Availability

The original contributions presented in the study are included in the article/**Supplementary Material**. Further inquiries can be directed to the corresponding author.
