# Supplementary material for: Entamoeba histolytica-induced NETs are highly cytotoxic on hepatic and colonic cells due to serine proteases and myeloperoxidase activities
Source: Front Immunol. 2024 Dec 2;15:1493946. doi: 10.3389/fimmu.2024.1493946 (PMC11646992; doi:10.3389/fimmu.2024.1493946)
Supplement: Supplementary file 1 [file Table1.docx]

Supplementary Material


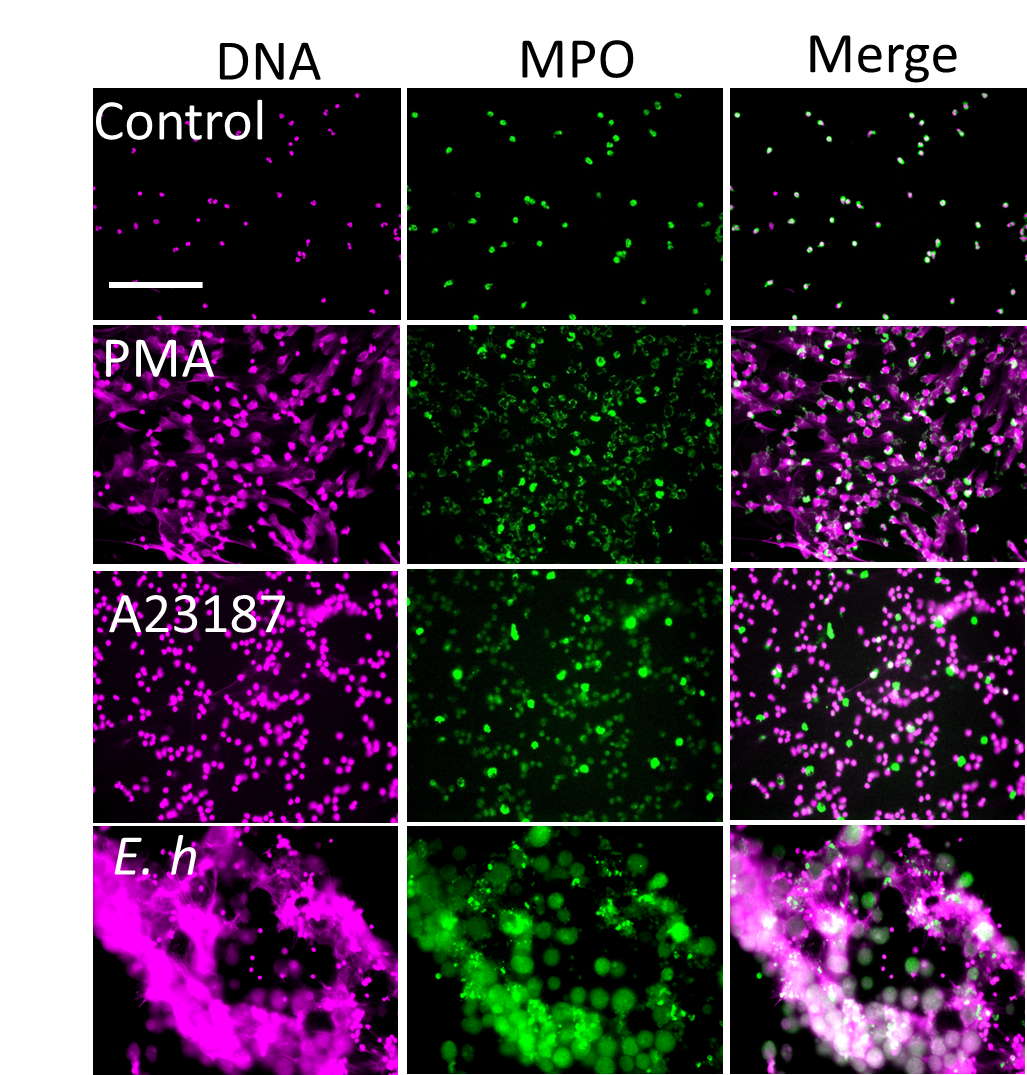


**Supplementary Figure 1. Determination of NET formation by *E. histolytica* trophozoites *in vitro* and its comparison with other NET stimuli.** Human neutrophils (PMN) (2 x 10^5^) were cultured in the presence of viable *E. histolytica* trophozoites (1 x 10^4^; neutrophil:amoeba ratio of 20:1), or stimulated with PMA (50 nM) or A23187 (10 μM). After 4 h of interaction, samples were fixed and treated with anti-MPO antibodies followed by FITC-conjugated secondary antibodies, and DNA was stained with DAPI. Images were obtained using a fluorescence microscope (Olympus BX51) at 40x magnification.


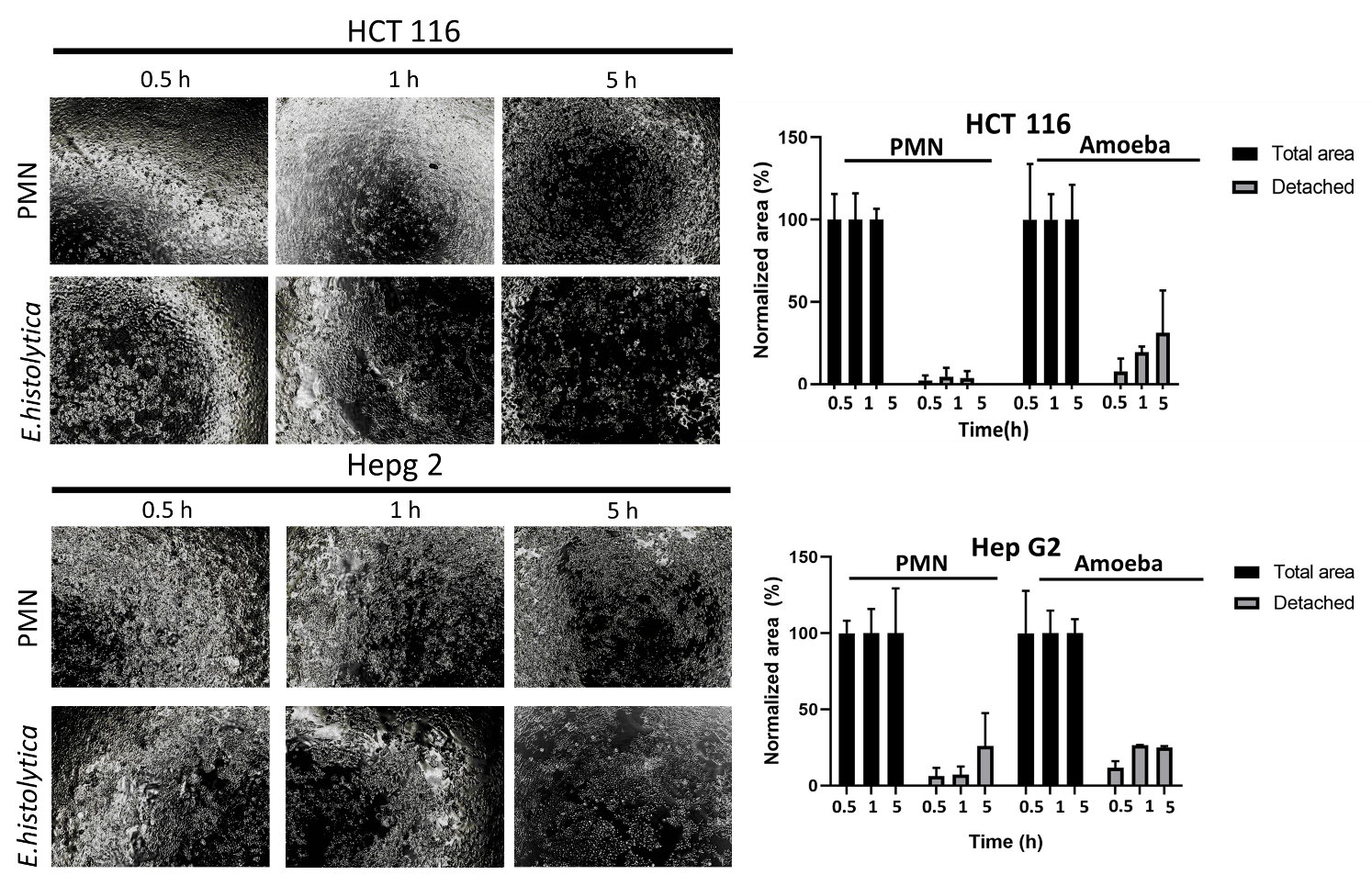


**Supplementary Figure 2. Effect of human neutrophils and *E. histolytica* trophozoites separately on HCT 116 colon and Hep G2 liver cell monolayers.** Cell monolayers with more than 90% confluence were exposed to 1 x 10^5^ neutrophils or 5 x 10^3^ trophozoites for 0.5, 1 and 5 h at 37°C with 5% CO_2_ atmosphere. Cultures were fixed with 4% v/v formaldehyde for 15 min and washed once with PBS. Samples were observed under an inverted microscope (Nikon) at 10X. Images obtained were processed with ImageJ software. The detachment area of the monolayer in the presence of amoebas or neutrophils was graphed at 0.5, 1, and 5 h.
